# Double Advantages of Nutrients and Biostimulants Derived from Sewage Sludge by Alkaline Thermal Hydrolysis Process for Agricultural Use: Quality Promotion of Soil and Crop

**DOI:** 10.1002/advs.202307793

**Published:** 2024-01-19

**Authors:** Jiahou Hao, Bingbing Li, Jiayi Tan, Yue Zhang, Xuejia Gu, Shuo Wang, Yun Deng, Xiaokai Zhang, Ji Li

**Affiliations:** ^1^ Jiangsu Key Laboratory of Anaerobic Biotechnology School of Environment & Ecology Jiangnan University Wuxi 214122 China; ^2^ College of Life Sciences Anhui Agricultural University Hefei 230036 China; ^3^ China Civil Engineering Society Water Industry Association Beijing 100082 China; ^4^ Heilongjiang Academy of Black Soil Conservation and Utilization Harbin 150086 China

**Keywords:** alkaline thermal hydrolysis, chemical fertilizer, enzyme activity, microbial community, sewage sludge, soil properties, total carbon emissions

## Abstract

Low‐carbon alkaline thermal hydrolysis of sewage sludge for the production of high‐quality plant‐growth‐promoting nutrients and biostimulants is a growing concern for sludge resource utilization in agriculture. Thus, this study aims to investigate functional characteristics and soil biochemical effects of sewage sludge‐derived nutrients and biostimulants (SS‐NB). The content of heavy metals in SS‐NB decreased by 47.39–100%, and an increase in soil protease, invertase, and soil nutrient utilization rates are observed in SS‐NB groups. SS‐NB substituted for chemical fertilizer increased the diversity and evenness of microbial community and reduced the abundance of the soil‐borne bacterial genus *Arthrobacter*. The dominant community of SS‐NB100 group is mainly enriched in *Microvirga*, *Ensifer, Novosphingobium*, *Bosea* and *Ellin6055*, which are principally beneficial symbiotic bacteria of plants and participated in C and N cycles. Moreover, SS‐NB reduced the accumulation of *Ktedonobacteria* and *Nitrosospira*, which are involved in the production of CO_2_ and N_2_O, and also enhanced the coordination of soil microorganisms with enzyme activities and nutrient utilization rate. In conclusion, the results suggest that SS‐NB exerts a positive effect on reducing greenhouse gas emissions and preventing soil‐borne diseases, and can further enhance collaboration with soil enzyme activity and soil nutrient utilization by stimulating soil microorganisms.

## Graphical abstract

Minimal research exists on the effects of SS‐NB on agricultural production. This research analyzed the low‐carbon emission characteristics and functional characteristics of sewage sludge‐derived nutrients and biostimulants, and revealed the beneficial effects of SS‐NB on soil enzyme activity, nutrient utilization rate and soil microorganisms and the interaction relationship, which is of great significance for sustainable agricultural production.

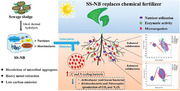

## 1 Introduction

With the continuous advancement of industrial development and urbanization, a large amount of CO_2_ is discharged into the environment, resulting in global environmental problems such as global warming and sea level rising.^[^ ^1^ ^]^ As one of the countries with high total carbon emissions in the world, it is urgent for China to implement carbon reduction strategy. The carbon emission of sewage treatment industry accounts for 1−2% of the total social emissions, and is an important field of carbon emission reduction.^[^ ^2^ ^]^ As an important part of sewage treatment process, it is necessary to study the carbon emission characteristics of sludge treatment. In the process of sludge treatment and disposal, a large number of chemicals and energy will be consumed, however, carbon compensation can also be formed through energy recovery or production of green products.^[^ ^3^ ^]^ Therefore, in the circumstance of “carbon peak and carbon emission reduction”, with the increasing scale of sewage treatment and the improvement of treatment standards, it has become an inevitable trend to achieve sustainable and healthy development to require the low‐carbon, harmless, and safe disposal of municipal sludge.

Fertilizers are essential agrochemicals in modern agriculture, which are widely used to substantially increase the crop productivity. Rapid population growth and the shortage of food supplies have motivated society to utilize larger quantities of chemical fertilizer. This is especially true for nitrogen from fertilizers, which is a most widely applied plant nutrient and a key contributor to agricultural productivity. Large quantity of fertilizer inputs and low fertilizer utilization ratio, resulting in the contamination of water environment and the reduction of soil quality. It has long been believed that N_2_O is emitted from residual chemical fertilizer by microorganisms in the soil,^[^ ^5^ ^]^ which further magnifies the greenhouse effect. In fact, several studies have highlighted the beneficial effects of organic fertilizers partially replacing chemical fertilizers on crop production, including improve soil structure to increase crop yield and quality, thereby contributing to sustainable agricultural development.^[^ ^6^, ^7^ ^]^ However, traditional organic fertilizers (animal manure, crop straw) were restricted from participating in agricultural production due to the low nutrient content, slow fertilizer efficiency, need of inactivate pathogenic bacteria through fermentation treatment, large amount of fertilizer application, and high transportation cost.^[^ ^8^, ^9^, ^10^ ^]^ Therefore, the development of high quality green inputs is the trend of sustainable agricultural development in the future.

At present, micromolecular plant‐growth nutrients and stimulants produced from sewage sludge are attracting increasing interest, as it is expected to enhance the fertilizing efficiency of land application.^[^ ^11^ ^]^ The annual output of sewage sludge has exceeded 70 million tons (80% moisture content) in China.^[^ ^12^ ^]^ Sludge contains a large number of nutrients, including N, P, K, proteins, polysaccharides, humic acids, nucleic acids, and lipids, among which protein content is the highest, accounting for 50% of the dry weight of bacterial cells.^[^ ^13^ ^]^ The beneficial components in sewage sludge can provide comprehensive nutrition for plant growth. Humic extracted from composted sewage sludge has been shown to promote root growth and proton pump activity in maize vesicles,^[^ ^14^ ^]^ and harmless sewage sludge is able to improve growth and yield of pepper by enhancing rhizosphere microorganism activity,^[^ ^15^ ^]^ indicating that the sewage sludge can be considered as biofertilizer or source of plant biostimulants. However, the toxic and harmful substances from sewage sludge are troubling in China. Although the stringent agricultural use standard of sludge has been issued in China, the agricultural use of sludge has been unable to be implemented due to the food safety and the limited capacity to decrease products toxicity, which greatly reduces the utilization effect of sludge resources in China. Thus, how to separate the beneficial products from harmful components is the core technical issue of sewage sludge agricultural application.

Alkaline thermal hydrolysis technology can accelerate activated sludge cell rupture, extracellular polymers flocs decomposition, and the degradation of organic matters, significantly increase the rate of protein dissolution.^[^ ^16^, ^17^ ^]^ The mixed solution by flash evaporation, solid–liquid separation, and enriched supernatant facilitates to get a green inputs called sewage sludge‐derived nutrient stimulant (SS‐NB) containing N, P, K, organic small molecules, peptides and free amino acids and biostimulants,^[^ ^18^ ^]^ which exert positive influences on regulating the nutrient uptake by plants and the tolerance to abiotic stress.^[^ ^19^, ^20^ ^]^ The technology of alkaline thermal hydrolysis has achieved the objectives of inactivation of all pathogenic bacteria, low heavy metal content, stabilization of refractory organic matter,

and creation of green agricultural inputs.^[^ ^21^, ^22^ ^]^ SS‐NB contain rich nutrients for plant growth, yet its effect mechanism on crop yield and soil physicochemical properties are unknown.

Soil microbial community plays a crucial role in soil nutrient cycling and crop growth.^[^ ^23^ ^]^ The functional capacity of soil microorganisms is closely related to enzyme activities involved in nutrient turnover.^[^ ^24^ ^]^ Soil invertase plays an important role in increasing soil soluble nutrients, which can be used to represent soil bioactivity. Enzymes related to the soil nitrogen cycle, including protease and urease, mainly participate in the mineralization and transformation of soil organic nitrogen, which is beneficial to improve the utilization rate of soil nitrogen. In addition, soil phosphatase, including acid phosphatase, neutral phosphatase, and alkaline phosphatase, is a kind of enzyme that catalyzes the mineralization of soil organic phosphorus compounds. Soil microorganisms are closely related to soil physicochemical properties and are directly affected by fertilizer characteristics.^[^ ^25^ ^]^ SS‐NB contain rich substrates for soil microbial metabolism, nevertheless the effects of SS‐NB on microbial characteristics and enzyme activities need to be further investigated.

Previous studies have demonstrated that soil properties, soil microorganisms, enzyme activities are greatly affected by fertilization management.^[^ ^26^ ^]^ In this study, SS‐NB is adopted to replace chemical fertilizer with the same amount of nitrogen, and the research objectives are 1) to account the carbon emissions of “alkaline thermal hydrolysis‐land application” and prove SS‐NB preparation by “alkaline thermal hydrolysis‐land application” is promising, 2) to evaluate the nutritional characteristics and safety of the SS‐NB, 3) to explore the effects of the SS‐NB replaces chemical fertilizer on soil physicochemical properties, enzyme activity, and microbial community composition, and 4) to analyze the relationships among soil nutrient properties, enzyme activity, and microbial community.

## 2 Results and Discussion

### 2.1 Total Carbon Emission of Typical Sludge Treatment and Disposal Process

#### 2.1.1 Total Carbon Emission of Deep Dewatering‐Dry Incineration‐Landfill Process

**Figure 1:**
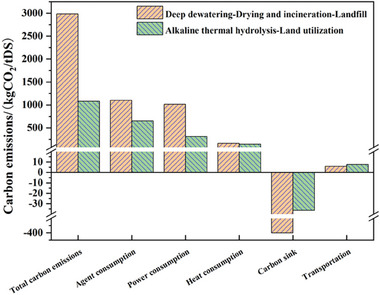
Total carbon emission in deep dewatering‐drying and incineration‐landfill and alkaline thermal hydrolysis‐land utilization process. tDS stands for Per ton of dry sludge.

The carbon emission performance of “deep dewatering‐dry incineration‐landfill” and “alkaline thermal hydrolysis‐land utilization” in entire sludge treatment and disposal process is shown in **Figure** **1**. The carbon emission of sludge with “deep dewatering‐drying incineration‐landfill” process is 2985.34 kg CO_2_/tDS^−1^, including direct carbon emissions, heat consumption, power consumption, chemicals consumption, and carbon emission caused by transportation and carbon sink. Carbon emissions from heat consumption, power consumption, chemicals consumption, and transportation together constitute the indirect carbon emissions of the total carbon emissions. Indirect carbon emission was 2288.63 kg CO_2_/tDS^−1^, accounting for 76.67% of the total carbon emissions. In the stage of deep dewatering, where quicklime and ferric chloride should be added, the indirect carbon emission caused by the consumption of the chemicals was 1103.00 kg CO_2_/tDS^−1^, accounting for 36.95% of the total carbon emission. In the process of deep dewatering, drying, incineration, and landfill, there are power consumption caused by equipment operation, and the corresponding carbon emission was 1016.36 kg CO_2_/tDS^−1^, accounting for 34.05% of the total carbon emission. The carbon emission caused by heat consumption and transportation was 163.43 and 5.84 kg CO_2_/tDS^−1^, respectively. In the sludge incineration process, there are direct emissions of greenhouse gases CH_4_ and N_2_O, which contributed to 1099.14 kg CO_2_/tDS^−1^ of direct carbon emissions, accounting for 27.58% of the total carbon emission. In addition, the heat generated by sludge incineration was reused in the sludge drying process, and the carbon sink obtained was 402.42 kg CO_2_/tDS^−1^.

#### 2.1.2 Total Carbon Emission of Alkaline Thermal Hydrolysis Process

The carbon emission of “alkaline thermal hydrolysis‐land utilization” process was merely 1080.64 kg CO_2_/tDS^−1^ (Figure 1), which can be divided into heat consumption, power consumption, chemicals consumption, and transportation. Indirect carbon emissions caused by heat consumption and power consumption were 143.36 and 313.33 kg CO_2_/tDS^−1^, accounting for 13.27% and 28.99% of the total carbon emission. In the process of “alkaline thermal hydrolysis”, quicklime and sulfuric acid were provided, and the indirect carbon emission caused by chemicals consumption was 652.64 kg CO_2_/tDS^−1^, accounting for 60.39% of the total carbon emission. The carbon sink of “alkaline thermal hydrolysis‐land utilization” are related to two parts: liquid protein fertilizer production and soil conditioner production. As for calculation of carbon sink, the nitrogen content of the two products is equal to that of chemical fertilizers, since they are adopted as the substitute for chemical fertilizers. Therefore, the carbon sink of “alkaline thermal hydrolysis‐land utilization” (36.34 kg CO_2_/tDS^−1^) accounts for 3.6% of the total carbon emissions, which should be deducted from the total carbon emissions. Carbon sink of alkaline thermal hydrolysis is less than that of heat recycling in sludge incineration stage, however, CH_4_, N_2_O, and other direct emissions of greenhouse gases were not produced. In addition, the carbon emission of transportation process is 7.65 kg CO_2_/tDS^−1^, accounting for 0.71% of the total carbon emission.

#### 2.1.3 Comparison of Total Carbon Emissions

**Figure 2:**
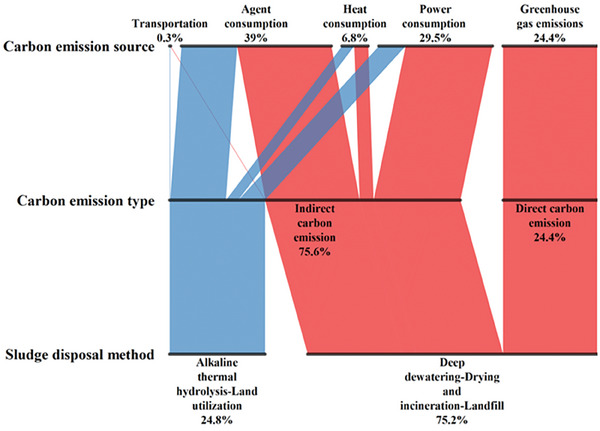
Total carbon emission source of deep dewatering‐drying and incineration‐landfill and alkaline thermal hydrolysis‐land utilization process.

The total carbon emission comparison (excluding carbon sinks) of the two sludge treatment and disposal methods is shown in **Figure** **2**. As chemicals were required in both sludge treatment and disposal processes, and the chemicals significantly contributed to carbon emission, indirect carbon emission caused by chemicals consumption accounts for the largest proportion among that from all carbon emission sources, accounting for 39% of the total carbon emission sources. The second largest total carbon emission was caused by power consumption (accounting for 29.5%). In the process of sludge treatment and disposal, a variety of equipment need to run continuously, resulting in a notable power consumption. Direct carbon emission accounted for 24.4% of the total carbon emission sources, while that from heat consumption and transportation accounted for only 6.8% and 0.3% of the total carbon emission. Additionally, indirect carbon emission played a dominant role in the total carbon emission (accounting for 75.6%), while direct carbon emission only accounted for 24.4%, and the main reason lies in the consumption of power and chemicals in sludge treatment and disposal. On the whole, the total carbon emission of “deep dewatering‐dry incineration‐landfill” was significantly higher than that of “alkaline thermal hydrolysis‐land utilization”, indicating that alkaline thermal hydrolysis would be a promising low‐carbon sludge treatment and disposal process.

### 2.2 SS‐NB Characteristics

#### 2.2.1 Nutrients and Biostimulants

SS‐NB is a high‐quality liquid fertilizer rich in plant‐growth‐promoting nitrogen‐containing nutrients and biostimulants from the sewage sludge. Alkaline thermal hydrolysis accelerates the sludge microorganism cell disruption, decomposition of extracellular polymer flocs, and the degradation of organic matters, thus more substrates are obtained. Nutritional characteristics of SS‐NB are shown in **Table** **1**. Continuous input of soil nutrients is an important way to maintain soil productivity, and the balance and availability of nutrients determine the yield and quality of crops.^[^ ^27^ ^]^ The conventional nutrients in SS‐NB include TN, TP, TK, NO_3_^−^‐N, NH_4_^+^‐N, AP, AK, and the content of N, P, and K is more than 10% (DM), which is much higher than 4% (DM) in the agricultural industry standard of organic fertilizer. In addition, nitrogen is an essential nutrient element for crops, and the TN (8.07 ± 0.14 [% DM]) in SS‐NB is higher than that in animal wastes (2.0–5.4 [% DM]) and typical anaerobic digestates (3.1–14.0 [% DM]).^[^ ^7^, ^28^, ^29^, ^30^ ^]^ Amino acids are easily absorbed by plants and have synergistic effects on nutrient cycling, which play important roles in plant growth and soil microbial metabolism, and calcium is one of the most important medium and trace elements in plant growth. In the SS‐NB, the protein and amino acids contents were 50.42 ± 0.85 and 5.42 ± 0.85 (% DM), respectively, and the content of calcium, iron, and magnesium reached 3.47 ± 0.23 (% DM). A large number of calcium, iron, and magnesium ions are easy to form chelates with amino acids, which can be quickly absorbed and utilized by plants,^[^ ^31^ ^]^ yet, these functions are not available in other organic fertilizers and chemical fertilizers. Moreover, the organic matter content was up to 21.21 ± 2.30 (% DM), including polysaccharides and small organic acids. Meanwhile, phytohormones and allelochemicals produced by sludge microorganisms, including indole‐3‐acetic acid (IAA), hydroxyphenyl acetic acids (HPAs), allelopathic of indolic derivatives (IDDs), and aromatic amino acids (AAAs) with contents of 1.91 ± 0.32, 1.83 ± 0.46, 2.81 ± 0.18, and 3.15 ± 0.39 (% DM), respectively, which were present in the form of trace organic acids and could be well utilized for plant growth.^[^ ^11^, ^18^ ^]^ In conclusion, SS‐NB contains abundant plant‐growth‐promoting nitrogen‐containing nutrients and biostimulants, and presents the function of regulating the nutrient uptake by plants. For functional regulation and agricultural development, SS‐NB demonstrates great advantages over other organic fertilizers and chemical fertilizers.

**Table 1:** Nutrient characteristics of SS‐NB and other organic fertilizers.

Parameters	SS‐NB	Animal wastes	Typical anaerobic digestates
Protein content	50.42 ± 0.85 (% DM)	ND	ND
Dry matter (DM)	58.77 ± 2.06%	9–26%	1.5–45.7%
pH	10.42 ± 0.20 (Diluted 800 times)	ND	7.3–9.0
Total nitrogen (TN)	8.07 ± 0.14 (% DM)	2.0‐5.4 (% DM)	3.1–14.0 (% DM)
Total phosphorus (TP)	1.09 ± 0.05 (% DM)	0.4–1.8 (% DM)	0.2–3.5 (% DM)
Total potassium (TK)	0.15 ± 0.00 (% DM)	1.2–3.2 (% DM)	1.9–4.3 (% DM)
Organic matter	21.21 ± 2.30 (% DM)	ND	36.7‐42.1 (% DM)
Nitrate (NO_3_^−^‐N)	1.04 ± 0.01 (% DM)	ND	ND
Ammonia nitrogen (NH_4_^+^‐N)	0.14 ± 0.00 (% DM)	ND	0.15–0.68 (% FM)
Available phosphorus (AP)	0.02 ± 0.00 (% DM)	ND	ND
Hydrolysis amino acid	5.42 ± 0.85 (% DM)	ND	ND
Calcium (Ca)	3.32 ± 0.21 (% DM)	1.1–5.5 (% DM)	0.01–0.023 (% FM)
Iron (Fe)	0.12 ± 0.02 (% DM)	ND	ND
Magnesium (Mg)	0.03 ± 0.00 (% DM)	ND	0.03–0.07 (% FM)
Indole‐3‐acetic acid (IAA)	1.91 ± 0.32 (% DM)	ND	ND
Hydroxyphenyl acetic acids (HPAs)	1.83 ± 0.46 (% DM)	ND	ND
Allelopathic of indolic derivatives (IDDs)	2.81 ± 0.18 (% DM)	ND	ND
Aromatic amino acids (AAAs)	3.15 ± 0.39 (% DM)	ND	ND

SS‐NB: sewage sludge‐derived nutrients and biostimulants

Animal wastes: Sheep manure, cow manure, pig manure, and chicken manure

Typical anaerobic digestates: cattle manure, livestock manure and agricultural residues, organic solid wastes and sewage sludge, dairy manure and biowastes, food wastes and landscape wastes, potato and sisal pulp wastes

ND: no data

#### 2.2.2 Heavy Metals

Heavy metals are the main limiting factors for sludge agricultural use. As shown in **Table** **2**, the average contents of Cu, Pb, Zn, Cd, Hg, As, Cr, and Ni from sludge in China are 283.5 ± 417.8, 78 ± 54.8, 925.7 ± 739.5, 5.8 ± 7.1, 2.8 ± 3.8, 15.7 ± 11.7, 136.9 ± 126.9 and 63.6 ± 79.2, respectively,^[^ ^32^ ^]^ which are significantly higher than the level of heavy metal from sludge in Shanxi.^[^ ^33^ ^]^ However, alkaline thermal hydrolysis of sludge may alleviate the heavy metals concerns because the increase of insoluble salts formed by hydrophobic materials and metals can be easily separated after centrifugation.^[^ ^18^ ^]^ The contents of Cu, Pb, Zn, Cd, Hg, As, Cr, and Ni in SS‐NB obtained by alkaline thermal hydrolysis process in Shanxi Province were 3.57 ± 1.21, 1.79 ± 0.29, 18.76 ± 0.96, 0.89 ± 0.62, 0.00 ± 0.00, 8.26 ± 1.13, 1.11 ± 0.93, and 27.36 ± 1.30 mg·kg^−1^, respectively. Compared with the untreated excess sludge in Shanxi Province, the respective contents of heavy metals Cu, Pb, Zn, Cd, Hg, As, Cr, and Ni in SS‐NB decreased by 97.80%, 95.48%, 93.33%, 54.82%, 100%, 44.30%, 99.27%, and 20.19%. Therefore, the quality of SS‐NB product is superior to the Grade A sludge product that listed in Control standards of pollutants in sludge for agricultural use (GB 4284‐2018). In addition, concentration of heavy metals in SS‐NB were far less than the content‐limits in many regulations, including water‐soluble fertilizer standard (NY1110‐2006), and organic fertilizer standard (NY/T 1978–2010) issued by the Ministry of Agriculture and Rural Affairs. In order to reduce the influence of SS‐NB on soil pH, it is generally diluted 800 times in practical agricultural application, therefore, the effects of SS‐NB on agricultural production safety is minimal.

**Table 2:** Metal content of sewage sludge, SS‐NB, limits of heavy metals in water soluble fertilizer (WSF), and organic fertilizer (OF).

Metals	Sewage sludge		SS‐NB	GB 4284‐2018	WSF	OF
	Mean values of China [mg kg^−1^]	Mean values of Shanxi [mg kg^−1^]	[mg kg^−1^]	limits for Grade A sludge products [mg kg^−1^]	Limit [mg kg^−1^]	
As	15.7 ± 11.7	14.83 ± 4.96	8.26 ± 1.13	< 30	10	15
Cd	5.8 ± 7.1	1.97 ± 3.55	0.89 ± 0.62	< 3	10	3
Cr	136.9 ± 126.9	152.26 ± 131.65	1.11 ± 0.93	< 500	50	150
Hg	2.8 ± 3.8	2.09 ± 1.95	< detection line	< 3	5	2
Pb	78 ± 54.8	39.56 ± 19.81	1.79 ± 0.29	< 3	50	50
Zn	925.7 ± 739.5	281.31 ± 336.66	18.76 ± 0.96	< 1200	ND	ND
Cu	283.5 ± 417.8	162.59 ± 105.80	3.57 ± 1.21	< 500	ND	ND
Ni	63.6 ± 79.2	34.28 ± 26.66	27.36 ± 1.30	< 100	ND	ND

GB 4284‐2018: Control standards of pollutants in sludge for agricultural use

Grade A sludge products: the types of agricultural land permitted include arable land, garden land and pasture land

ND: no data

### 2.3 Responses of Soil Nutrient and Enzyme Activities

#### 2.3.1 Soil Fertility and Crop Yield

Partial substitution of chemical fertilizer with SS‐NB greatly influenced the physical and chemical properties of soil. The characteristics of pH, electric conductivity (EC), organic matter, and soil C/N ratio in different fertilization groups are shown in Table S1 (Supporting Information). The increase of SS‐NB caused a pH increase of soil. The soil pH of SS‐NB100 group was the highest, reaching 8.21 ± 0.02, which was equivalent to that of initial soil (pH = 8.29 ± 0.01). For the SS‐NB0, soil pH decreased to 7.72 ± 0.14, however, in the SS‐NB groups, the lowest pH value only decreased to 7.92 ± 0.10. The results suggest that SS‐NB can alleviate the problem of soil acidification caused by chemical fertilizer and would not lead to a sudden increase in soil pH. Soil organic matter and cation content are closely related to soil fertility, soil organic matter, and electrical conductivity decreased in all groups after harvest. In the fertilization group, organic matter, and electrical conductivity increased along with the increase of SS‐NB. The experimental results showed that SS‐NB could not only provide crops with nutrients, but also timely supplement more soil organic matter and cation contents compared with chemical fertilizer.

Soil physical and chemical properties are closely related to promoting plant production and maintaining soil quality.^[^ ^34^ ^]^ The fresh weight (6.28 ± 1.68 g) and dry weight (0.49 ± 0.05 g) of *Pakchoi cabbages* in SS‐NB100 were 1.9 and 1.3 times higher than that in CK, respectively, while fresh weight and dry weight in SS‐NB50 and SS‐NB25 were further increased, and the yield was approximately equivalent to that in SS‐NB0 (Figure S1, Supporting Information), the application SS‐NB improved *Pakchoi cabbages* yield, and the effect was better when combined with fertilizer.^[^ ^35^ ^]^ The annual mineralization rate of organic fertilizer is 18–55%, and in order to simulate the actual organic production capacity, the application amount of organic fertilizer is usually twice that of inorganic fertilizer.^[^ ^36^ ^]^ In this experiment, SS‐NB100 can reach 84% of SS‐NB0 *Pakchoi cabbages* fresh weight yield under the same nitrogen level, indicating that the nutrients in SS‐NB can be easily absorbed and utilized by *Pakchoi cabbages*.

#### 2.3.2 Soil Nutrient Cycle

**Figure 3:**
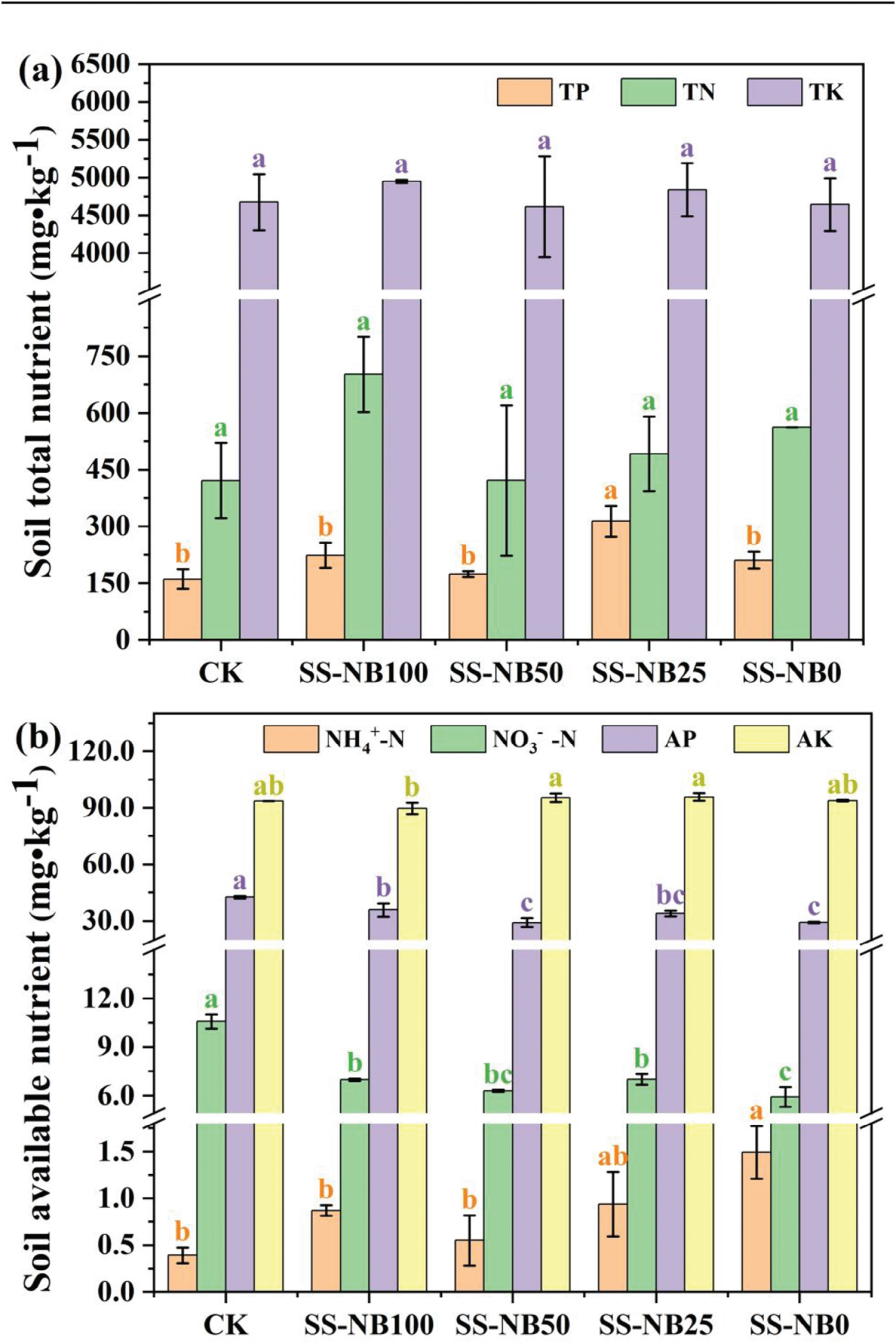
Effects of different proportions of SS‐NB replacement fertilizer on soil total nutrients and available nutrients. TN: total nitrogen, TP: total phosphorus, TK: total potassium, NO^3−^‐N: nitrate, NH^4+^‐N: ammonium, AP: available phosphorus, AK: available potassium. CK stands for no nitrogen fertilizer application; SS‐NB100, SS‐NB50, SS‐NB25, and SS‐NB0 are 100%, 50%, 25%, and 0% substitution of chemical fertilizer by SS‐NB, respectively.

Nutrient content and availability are important indices to evaluate soil quality. At the end of the experiment, the characteristics of total nutrition and available nutrition were shown in **Figure** **3**. In general, the contents of TN, TP, and TK in fertilization groups were correspondingly increased. Due to the high soil background value of TK, the increase of TK content was not obvious. In SS‐NB25, TP increased most significantly, reaching 95.5%. TN, with higher element nitrogen content in SS‐NB, experienced the highest increase in SS‐NB100, up to 66.7%. For available nutrients, compared with CK, AK in fertilization group had no notable change, AP slightly decreased, while NO_3_^−^‐N significantly decreased, reaching 44.1%. However, the content of NH_4_^+^‐N showed completely different results, with a significant increase in all fertilization groups. In SS‐NB0 group, NH_4_^+^‐N content increased as much as 2.82 times. SS‐NB contained total nutrients (TN, TP, TK) and available nutrients (AP, NO_3_^−^‐N). Total nutrients can be absorbed by plants only through decomposition and transformation, while available nutrients can be absorbed directly by plants, thus resulting in the aggregation of total nutrient content and the reduction of available nutrient content in soil. The increase in NH_4_^+^‐N content may be due to the degradation of proteins contained in SS‐NB groups (SS‐NB100, SS‐NB50, and SS‐NB25) and conversion of urea in SS‐NB0 group.

In order to further explore the influence of SS‐NB on soil nutrient status, the soil nutrient utilization rates (TN, TK, AK, AP, NO_3_^−^‐N) of the fertilization group are calculated (**Table** **3**). The highest utilization rate of TN (58%), TK (19%), AK (37%), AP (50%), and NO_3_^−^‐N (81%) in SS‐NB fertilization group was higher than that in CK group and SS‐NB0 group, and the utilization rate of AK and NO_3_^−^‐N increased along with the increase of SS‐NB. The experimental results clearly showed that SS‐NB caused changes in soil nutrient characteristics. SS‐NB introduced a large number of comprehensive nutrients, and with the participation of soil microbial and enzyme activities, promoting the mineralization efficiency of soil nutrients and the nutrient absorption efficiency of plants, and ultimately enhancing the utilization rate of soil nutrients. Similar conclusions showed that soil quality was improved by organic matter input and mineralized nutrient supply, which was beneficial to finally increase plant productivity.^[^ ^37^ ^]^

**Table 3:** Soil nutrient utilization efficiency.

Treatment	TN	AK	TK	AP	NO_3_^−^‐N
CK	54%	23%	18%	27%	7%
SS‐NB100	36%	37%	13%	39%	81%
SS‐NB50	58%	28%	19%	50%	73%
SS‐NB25	49%	25%	15%	42%	60%
SS‐NB0	38%	23%	18%	50%	48%

#### 2.3.3 Soil Enzymes Activities

**Figure 4:**
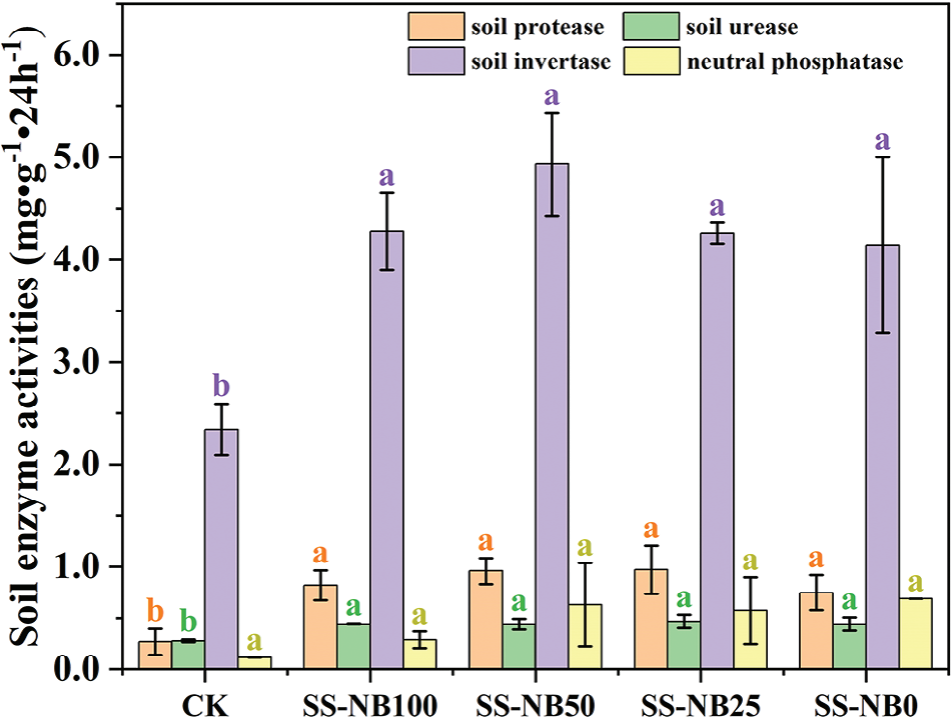
Effects of different proportions of SS‐NB replacement fertilizer on soil protease, urease, invertase, and neutral phosphatase. CK stands for no nitrogen fertilizer application; SS‐NB100, SS‐NB50, SS‐NB25, and SS‐NB0 are 100%, 50%, 25%, and 0% substitution of chemical fertilizer by SS‐NB, respectively.

Soil enzymes play vital roles in soil nutrient cycling and metabolic processes,^[^ ^38^ ^]^ and the activity characteristics of soil protease (PRO), urease (URE), invertase (INV), and neutral phosphatase (NEP) in fertilization groups was depicted in **Figure** **4**. Compared to the CK group, the activities of soil protease, urease, invertase, and neutral phosphatase in fertilization group were significantly increased, ranging from 58.4% to 500%. The activities of soil protease and invertase in SS‐NB groups (SS‐NB100, SS‐NB50, and SS‐NB25) were higher than those in SS‐NB0 group, and the maximum increase rate was up to 29.5%. However, soil neutral phosphatase activity in SS‐NB groups (SS‐NB100, SS‐NB50, and SS‐NB25) was lower than that in SS‐NB0 group (0.69 ± 0.00 mg g^−1^·24 h), especially in SS‐NB100 group, the soil neutral phosphatase was only 0.29 ± 0.08 mg g^−1^·24 h. For soil urease, there was no significant difference among fertilization groups. The experimental results are roughly consistent with the conclusion that organic manure and chemical fertilizer resulted in the increase of soil enzyme activity.^[^ ^39^ ^]^ Proteases are involved in the transformation of protein‐based organisms and invertases play important roles in the transformation of soluble nutrients.^[^ ^40^ ^]^ SS‐NB contains a large amount of protein and soluble nutrients, which may be responsible for the higher activities of protease and invertase in SS‐NB group. Phosphatase activity is an index to evaluate the direction and intensity of soil phosphorus biotransformation and is related to soil available phosphorus and pH value,^[^ ^41^ ^]^ and soil neutral phosphatase activity may be sensitive to excessive SS‐NB. These results suggest that the proper ratio of SS‐NB to chemical fertilizer has the potential to improve soil enzyme activities.

### 2.4 Responses of Soil Microbial Community

#### 2.4.1 Microbial Diversity

A total of 548 280 effective sequences and 4109 operational taxonomic units (OTUs) were generated for 18 samples. In order to evaluate the effect of substituting SS‐NB for chemical fertilizer (CF) on soil microbial community diversity, the alpha diversity of soil samples is examined in all groups, represented by ACE, Chao, Simpson, and Shannon indices (Table S2, Supporting Information). ACE and Chao were adopted to analyze the abundance of microbial community. In general, the abundance of microbial community was reduced after fertilization, while the abundance of microbial community in SS‐NB groups was greater than that in CF group. Simpson and Shannon indices were used to analyze the diversity and evenness of microbial community. Fertilization reduced the diversity and evenness of microbial community, and the addition of SS‐NB increased the diversity and evenness of microbial community compared with the application of CF (SS‐NB0). A similar conclusion was reached that the alpha diversity of soil microbial community was reduced by the application of chemical fertilizer and manure.^[^ ^42^, ^43^ ^]^

**Figure 5:**
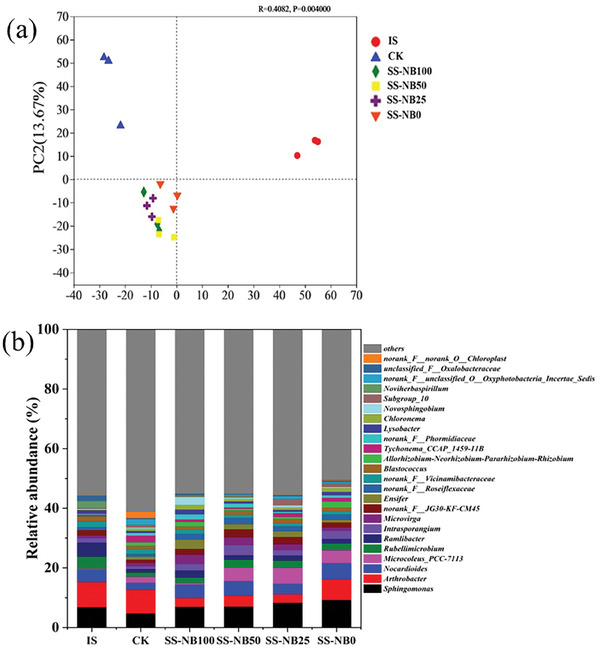
a) Soil microbial community dissimilarity by applying principal component analysis (PCA) on OTU level; b) soil microbial community structures at genus level. IS stands for initial soil; CK stands for no nitrogen fertilizer application; SS‐NB100, SS‐NB50, SS‐NB25, and SS‐NB0 are 100%, 50%, 25%, and 0% substitution of chemical fertilizer by SS‐NB, respectively.

Overall dissimilarities of the soil bacterial communities were shown in principal component analysis (PCA) at the OTU level, and high reproducibility in triplicate samples were obtained. Apart from the initial soil (IS) and CK, soil bacterial communities of the SS‐NB100, SS‐NB50, SS‐NB25, and SS‐NB0 were clustered together (**Figure** **5**a), suggesting that the addition of fertilization significantly affected the dissimilarity of bacterial communities. Based on the dispersion of SS‐NB100, SS‐NB50, SS‐NB25, and SS‐NB0 on the PC1 axis, as shown in Figure S2 (Supporting Information), the median of dispersion is −7.4516, −6.8372, −9.5726, and −1.1982, respectively. Considerable similarity was revealed among the bacterial communities of SS‐NB100, SS‐NB50, SS‐NB25, and different from CF (SS‐NB0). The results showed that SS‐NB replacement of chemical fertilizer also caused significant changes in bacterial community.

#### 2.4.2 Microbial Functional Composition

Twenty‐four of the most abundant OTUs, with relative abundance ≥ 1.5% in all soil samples, were detected in bacterial community analysis (Figure 5b). At the phylum level (Figure S3, Supporting Information), *Proteobacteria*, *Actinobacteriota*, *Cyanobacteria*, and *Acidobacteriota* were dominant in the IS, CK, SS‐NB100, SS‐NB50, SS‐NB25, and SS‐NB0. *Proteobacteria* played an significant role in fixing atmospheric nitrogen to ammonia and providing it to the host plant,^[^ ^44^ ^]^ and presented relatively higher abundance in SS‐NB100 (49.0%), SS‐NB50 (38.5%), and SS‐NB25 (41.2%) than in SS‐NB0 (37.7%). The most abundant OTU in the SS‐NB100, SS‐NB50, SS‐NB25, and SS‐NB0, was assigned to genus *Sphingomonas* belonging to *Proteobacteria*, which have been found to be a keystone genus in healthy soils, and was also significantly associated with plant pathogen suppression.^[^ ^45^ ^]^ *Actinobacteriota* have similar abundance in all groups, which were identified as critical for maintaining potential soil multi‐functionality.^[^ ^46^ ^]^

*Arthrobacter*, a widespread soil‐borne bacterial genus belonging to *Actinobacteriota*,^[^ ^47^ ^]^ was enriched at a lower relative abundance in SS‐NB100 (2.95%), SS‐NB50 (3.61%), and SS‐NB25 (2.79%) than in SS‐NB0 (7.01%), which may be reduced by SS‐NB application instead of chemical fertilizer. Similar conclusion was drawn that compost process was facilitated to reduce the relative richness of *Arthrobacter* in soil.^[^ ^48^ ^]^ *Nocardioides*, as the identified genera in *Actinobacteriota* that may participate a role of plant growth‐promoting microbes and organic matter decomposers,^[^ ^49^ ^]^ were at a greater abundance in SS‐NB100, SS‐NB50, SS‐NB25, and SS‐NB0 than in CK. *Cyanobacteria* improve soil fertility through carbon and nitrogen cycles and balancing mineral nutrition in the soil.^[^ ^50^ ^]^ *Microcoleus_PCC_7113*, as a part of *Cyanobacteria*, was enriched in SS‐NB50 (4.56%), SS‐NB25 (5.45%), and SS‐NB0 (4.29%) and decreased in SS‐NB100 (0.56%). Compared with SS‐NB0, although a lower abundance of Microcoleus_PCC_7113 affiliated with *Cyanobacteria* was observed in SS‐NB100 (0.56%), cycle of soil nutrient elements may not have been impeded due to the higher relative abundance of genus level assigned to nitrogen‐fixing, symbiotic, beneficial bacteria (*Microvirga*, *Ensifer*, *Bosea*),^[^ ^51^, ^52^, ^53^ ^]^ and organic decomposition bacteria (*Novosphingobium*, *Ellin6055*)^[^ ^54^, ^55^ ^]^ from bacterial community heatmap analysis (Figure S4, Supporting Information). The results showed that SS‐NB instead of chemical fertilizer could create higher abundance of nitrogen‐fixing and beneficial functional microorganism, and significantly reduce the abundance of soil‐borne bacteria. The application of organic fertilizer instead of chemical fertilizer increased the number of nitrogen‐fixing bacteria, phospho‐increasing bacteria, and potassium‐increasing bacteria, and was conducive to reduce the abundance of soil‐borne diseases *Ralstonia* and *Fusarium*.^[^ ^56^, ^57^, ^58^ ^]^

**Figure 6:**
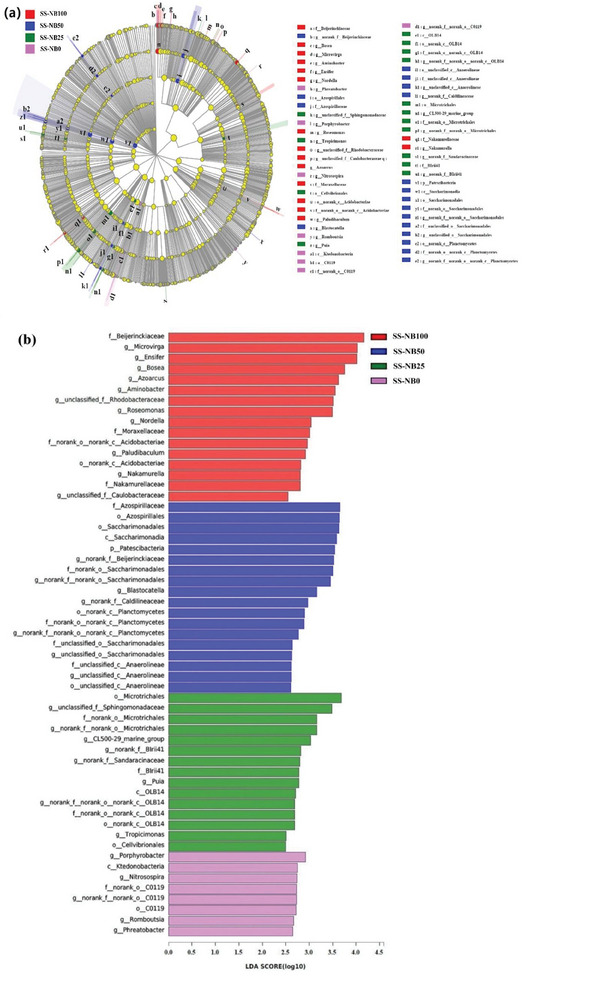
Significance and differential analysis of microbial communities in SS‐NB100, SS‐NB50, SS‐NB25, and SS‐NB0. a) Cladogram indicating the significant taxonomic features among microbial taxa; b) Histogram of linear discriminant analysis (LDA) effect Size (LEfSe) showing taxa with log10 (LDA scores) ≥ 2.5.

The cladogram (**Figure** **6**a) and LDA Effective Size (LEfSe) histogram (Figure 6b) further identified the different microbial communities in fertilization groups. A total of 57 taxa had LDA scores ≥ 2.5, and included 16 taxa in the SS‐NB100, 18 taxa in the SS‐NB50, 15 taxa in the SS‐NB25, and 8 taxa in the SS‐NB0. The top three most abundant bacteria taxa in the SS‐NB100 were *Beijerinckiaceae*, *Microvirga*, and *Ensifer*, which mainly have nitrogen fixation function.^[^ ^52^, ^59^ ^]^ *Azospirillaceae*, *Azospirillales*, and *Saccharimonadales*, the top three most important bacteria taxa in the SS‐NB50, are able to promote plant growth and phosphatase activity and participate in nitrogen cycling.^[^ ^60^, ^61^ ^]^ The top abundant bacteria communities in the SS‐NB25 included *Microtrichales* and *Sphingomonadaceae*, which were vital to hydrolyze and utilize complex organic matters.^[^ ^62^, ^63^ ^]^ The results showed that the addition of SS‐NB enhanced the degradation of soil organic matter, enzyme activity, and nutrient cycling. However, in the SS‐NB0, the top abundant bacteria communities including *Ktedonobacteria* and *Nitrosospira* can oxidize hydrogen and carbon monoxide, and promote CO_2_ and N_2_O emissions.^[^ ^64^, ^65^ ^]^ It has been reported that nitrogen input is related to greenhouse gas emissions,^[^ ^66^ ^]^ under the same nitrogen input, SS‐NB instead of chemical fertilizer had potential functions in preventing the release of soil CO_2_ and N_2_O through reducing greenhouse gas‐associated microorganisms.

#### 2.4.3 SS‐NB Enhanced Collaboration

**Figure 7:**
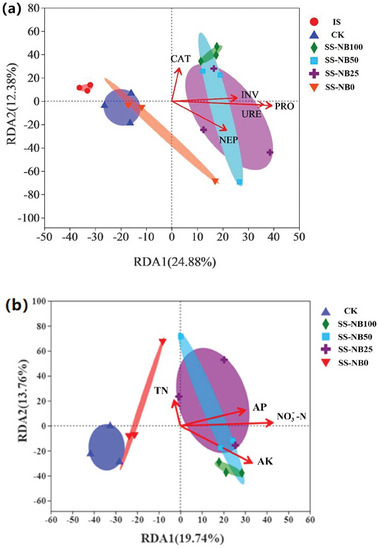
Redundancy analysis (RDA) between the soil enzyme activities a), soil nutrient availability b) and soil microbial taxa at the OTU level. IS stands for initial soil; CK stands for no nitrogen fertilizer application; SS‐NB100, SS‐NB50, SS‐NB25, and SS‐NB0 are 100%, 50%, 25%, and 0% substitution of chemical fertilizer by SS‐NB, respectively.

*Soil Carbon and Nitrogen Cycling*: Soil extracellular enzymes are synthetized and secreted primarily by soil microorganisms, and are involved in various biochemical processes in soil, therefore, soil enzyme activity is usually used to evaluate soil quality. Redundancy analysis (RDA) revealed the correlations between soil enzyme activities and bacterial community composition at the OTU level (**Figure** **7**a). The soil enzyme activity could account for 37.26% of bacterial variations, which Axis 1 of the RDA plot explained roughly 24.88% of the variation, while axis 2 explained a further 12.38%. The bacterial communities of IS, CK, and SS‐NB0 mostly clustered in the negative half of axis 1 and were negatively correlated with catalase (CAT), INV, PRO, URE, and NEP activity, while the bacterial communities of SS‐NB100, SS‐NB50, and SS‐NB25 clustered in the positive half of axis 1 and were significantly positively correlated with CAT, INV, PRO, URE, and NEP activity. From Figure S5 (Supporting Information), it was also observed that INV, PRO, URE, and NEP activity was positively correlated with the abundance of *Nitrospirota* and *Chloroflexi*, which were closely related to soil carbon and nitrogen cycling.^[^ ^67^, ^68^ ^]^ In summary, these findings suggest that the addition of SS‐NB strengthens the relationship between microbial and enzyme activity in soils, particularly by microbes that most readily respond to promote soil carbon and nitrogen cycling.

*Soil Nutrient Utilization Rate*: Soil microorganisms are both the driving force behind the conversion and cycling of soil nutrients and the reservoir of effective plant nutrients in the soil. RDA further explored the correlations between soil nutrient utilization rate and bacterial community composition at the OTU level (Figure 7b), the bacterial communities of SS‐NB100, SS‐NB50, and SS‐NB25 were significantly positively correlated with TP, AK, NO_3_^−^‐N utilization rate, yet CK and SS‐NB0 were opposites. In SS‐NB100 and SS‐NB50 groups, TN utilization rate was negatively correlated with the soil bacterial microbial community. However, it showed a strong positive correlation in SS‐NB25 and SS‐NB0, which increased gradually along with the increase in the proportion of chemical fertilizer. The experimental results show that SS‐NB provides plants with readily available nutrients, which does not result in a significant increase in soil available nutrients (Figure 3b), but more importantly, SS‐NB stimulates microorganisms and enhances the collaboration between soil microorganisms and nutrient cycling. This eventually led to higher nutrient utilization in SS‐NB100, SS‐NB50, and SS‐NB25 (Table 3). It seems that the higher the chemical fertilizer nitrogen (TN) content, the stronger the association with soil microorganisms. In the SS‐NB0 group, LEfSe showed a significant aggregation of N_2_O producing microorganisms, which may be responsible for the relatively high addition of chemical fertilizer.

*Soil Productivity Improvement by SS‐NB*: In this study, it was also found that EC, pH, TN, NO_3_^−^‐N, AP, and AK were negatively correlated with soil INV, PRO, URE, and NEP activity (Figure S6, Supporting Information). These results suggest that soil nutrient dynamics are negatively correlated with soil enzyme activity. Soil microorganisms, as the central hub of soil ecological functions, are concerned with soil quality. SS‐NB may affect soil productivity in the following ways. First, the readily available nutrients in SS‐NB can directly affect soil quality and plant yield. Furthermore, SS‐NB contains amino acids and other biostimulants, which provide indispensable substrates for improving soil enzyme activity. In addition, SS‐NB also provides nitrogen‐rich nutrient substrates to soil microorganisms, driving material exchange along with soil enzyme activity and nutrients, which ultimately benefits plants.

## 3 Conclusion

The carbon emissions in the process of “alkaline thermal hydrolysis–land use” of sludge are relatively low, chemical consumption accounts for 60.39% of the total carbon emissions, and the carbon sink is 36.34 kg CO_2_/tDS^−1^. Indirect carbon emissions caused by chemical consumption and electricity consumption are the main factors for the increase of total carbon emissions. The substitution of SS‐NB for chemical fertilizer affected soil biochemical characteristics. The results showed that SS‐NB replaced chemical fertilizers to supplement soil organic matter and cation content, reduce soil acidification, improve soil TN, NO_3_^−^‐N, AK, and AP utilization rate, significantly change soil microbial community structure, and promote the bacteria diversity and evenness. Additionally, the application of SS‐NB enhanced the coordination of soil microorganisms with enzyme activities (CAT, INV, PRO, URE, NEP) and nutrient availability (TP, AK, NO_3_^−^‐N) in soil. Compared with SS‐NB0, the dominant community of SS‐NB100 was more enriched with *Microvirga*, *Ensifer*, *Novosphingobium*, *Bosea*, and *Ellin6055*, which were main beneficial symbiotic bacteria of crop growth and participated in carbon and nitrogen cycles. SS‐NB replacement for chemical fertilizer also reduced the abundance of the soil‐borne bacterial genus *Arthrobacter*. LEfSe analysis showed that the application of SS‐NB reduced soil CO_2_ and N_2_O emissions through microbial action (*Ktedonobacteria* and *Nitrosospira*). This study demonstrated enormous benefits of SS‐NB as green inputs in improving soil quality and reducing carbon emission.

## 4 Experimental Section

### Soil and Experimental Materials

Experimental soil was obtained from the Land management office of Jiangnan University (Wuxi, China), and the surface soil (0–20 cm) was collected in July 2021 (site N, 120°27′E, and 31°58′N). After air drying, the soil was screened with 8 mm screen for pot experiment. The experimental crop is *pakchoi cabbage* (Beijing Aohua Agriculture Co. Ltd.), and the analytical grade urea is used as chemical fertilizer. In addition, the nutritive biostimulants (NB) were extracted from sewage sludge (SS) of municipal sewage treatment plant (the proportion of domestic sewage is 80%) via alkaline thermal hydrolysis process (Shanxi Jinlian Environmental Technology Co., Ltd., China). Phytohormones and allelopathic substances in SS‐NB were extracted according to previous reports on dissolved organic samples.^[^ ^11^, ^20^ ^]^ Details on target substance extraction, GC/TOF‐MS determination, and concentration quantification are provided in the supplemental materials, Text S1 (Supporting Information).

### Experimental Design

The pot experiments consisted of the following five groups: 1) CK (no nitrogen fertilizer application); 2) SS‐NB100 (substitution of chemical fertilizer with SS‐NB at 100%); 3) SS‐NB50 (substitution of chemical fertilizer with SS‐NB at 50%); 4) SS‐NB25 (substitution of chemical fertilizer with SS‐NB at 25%); and 5) SS‐NB0 (substitution of chemical fertilizer with SS‐NB at 0%), each group was conducted in triplicates. In addition, SS‐NB100, SS‐NB50, and SS‐NB25 were classified as the SS‐NB group, SS‐NB0 group was sorted as the chemical fertilization (CF) group, and SS‐NB100, SS‐NB50, SS‐NB25, and SS‐NB0 were classified as the fertilization group. *Pakchoi cabbages* were grown in plastic pots containing 500 g dry soil, each of which was 10 × 8 × 6.7 cm in length, width, and height, and four seedlings were kept in each pot after one week. SS‐NB and urea were diluted 400 and 500 times, respectively, and applied to the soil several times, maintaining the same amount of water and TN in each group. The *Pakchoi cabbage* is sown on July 30, 2021, and harvested on September 15, 2021, with a 47‐day growing period and temperature range between 23 and 35 °C.

### Soil Sampling

Potting soil (0–5 cm) was collected from each pot experiment at the harvest time of *Pakchoi cabbage* in five‐point sampling method, and mixed to form a composite sample. The collected soil samples were separated into two fractions: one portion was immediately stored at −80 °C for Illumina sequencing, and the other was air‐dried for the analysis of soil physicochemical properties and enzymatic activity.

### Soil Properties and Enzymatic Activities

Soil pH value was monitored using a pH meter (STARTER3100, OHAUS Instruments, Shanghai, China) at a soil:water ratio of 1:5. Soil electrical conductivity (EC) was monitored using Portable conductivity meter (Multi 3630 IDS, WTW Instruments, Munich, Germany). Total nitrogen (TN) was measured via the Automatic Kjeldahl apparatus (K9840, Hanon Instruments, Dezhou, China). Nitrate (NO_3_^−^‐N) and ammonium (NH_4_^+^‐N) contents were assessed following the extraction of fresh soil.^[^ ^69^ ^]^ Additionally, total phosphorus (TP) and available phosphorus (AP) were quantified spectrophotometrically (DR6000, HACH, Loveland, USA) using the molybdenum blue colorimetric analysis. Total potassium (TK) and available potassium (AK) were measured via inductively coupled plasma emission spectrometer (ICP), and soil C/N ratio was calculated using elemental analyzer. Soil urease activity was quantified after incubating 5 g of soil with 10 mL of a 10% urea solution and 20 mL citrate buffer solution for 24 h at 37 °C, and the concentration of ammonia nitrogen indicates urease activity. In addition, soil neutral phosphatase activity was determined by benzene disodium phosphate colorimetric method, and the content of free phenol indicates the phosphatase activity. Soil invertase and catalase (CAT) activities were determined by 3, 5‐dinitrosalicylic acid colorimetric method and permanganate titration, respectively. Furthermore, soil protease activity was measured by the colorimetry method, represented by glycine content.

### Microbial Community Analysis

Soil samples from the pot experiments, including the initial soil (IS), CK, SS‐NB100, SS‐NB50, SS‐NB25, SS‐NB0 were collected for microbial analysis. Microbial DNA was extracted from soil samples using the E.Z.N.A. soil DNA Kit (Omega Bio‐tek, Norcross, GA, U.S.) according to manufacturer protocols. Illumina MiSeq sequencing was conducted for the samples at the Sequencing Services Facility at the Majorbio Bio‐Pharm Technology Co. Ltd. (Shanghai, China). Briefly, PCR amplification was performed using the primer 338F (5′‐ ACTCCTACGGGAGGCAGCAG‐3′) and 806R (5′‐GGACTACHVGGGTWTCTAAT‐3′) for Bacteria. Amplicons of the PCR reactions were purified and then sequenced using Illumina MiSeq PE300 platform (Illumina, San Diego, USA). The raw reads were deposited into the NCBI Sequence Read Archive (SRA) database as BioProject PRJNA789999.

Raw fastq files were quality‐filtered by Trimmomatic and merged by FLASH. Operational taxonomic units (OTUs) were clustered with 97% similarity cutoff using UPARSE (version 7.1 http://drive5.com/uparse/) with a novel “greedy” algorithm that performs chimera filtering and OTU clustering simultaneously. The taxonomy of each 16S rRNA gene sequence was analyzed by RDP Classifier algorithm (http://rdp.cme.msu.edu/) against the Silva (SSU123) 16S rRNA database using confidence threshold of 70%. Microbial community composition and differences analysis, principal component analysis (PCA), and correlation analysis of environmental factors were performed using the online platform of Majorbio Cloud Platform (www.majorbio.com).

### Carbon Emission Calculation

The carbon emission calculation method is evolved in the “Guidelines for National Greenhouse Gas Inventory” of Intergovernmental Panel on Climate Change (IPCC), which stipulates that the carbon emission in the whole process of sludge treatment and disposal includes direct carbon emission, indirect carbon emission, and carbon sink.^[^ ^70^ ^]^ Direct carbon emission refers to CH_4_ and N_2_O generated during the operation of sludge treatment and disposal. In terms of calculation, other greenhouse gases should be calculated as CO_2_ equivalent, and included in the carbon emission according to the Global Warming Potential (GWP) provided by IPCC. It should be pointed out that CO_2_ from sludge biodegradation or incineration is not included in the calculation according to the IPCC guidelines.^[^ ^71^ ^]^ Indirect carbon emission refers to the carbon emission generated by chemicals and energy consumed in sludge treatment and disposal process, including chemicals consumption, power consumption, heat consumption, and fuel consumption in transportation process. Carbon sinks are energy recovery or energy output from products used to replace raw materials or fuels. Alkaline thermal hydrolysis can produce protein liquid fertilizer and biostimulants to replace chemical fertilizers, and the heat generated by the incineration process can be reused in the drying stage.^[^ ^72^ ^]^ The carbon emission accounting boundary is shown in Figure S7 (Supporting Information). In this study, the carbon emission accounting is based on the treatment and disposal of 1 t dry sludge (20 tons of sludge with 95% water content). Table S3 (Supporting Information) presents the energy consumption and chemicals consumption parameters of each process link, and the data are all from the process operation parameters of the practical sludge treatment and disposal project. Table S4 (Supporting Information) lists the CO_2_ emission factors, and the data are from the IPCC checklist guidelines or literature.^[^ ^73^, ^74^, ^75^, ^76^ ^]^

### Statistical Analysis

Data are expressed as the averages of three replicates for each treatment. Pearson's correlation test was used to evaluate correlations among the different parameters, and a one‐way analysis of variance (ANOVA) with the Fisher LSD test was used to determine significant differences at a confidence level of *p* < 0.05. Linear discriminant analysis (LDA) effect size (LEfSe) was adopted to perform linear discriminant analysis (LDA) on samples according to different grouping conditions based on their taxonomic composition, in order to identify communities or species that have a significant differential impact on sample partitioning (https://huttenhower.sph.harvard.edu/lefse/). The R language vegan package (vsesion2.4.3) was used for Redundancy analysis (RDA) analysis and mapping.

## Conflict of Interest

The authors declare no conflict of interest.

## Supporting information

Supporting Information

## Data Availability

The data that support the findings of this study are available from the corresponding author upon reasonable request.
