# Supplementary material for: Double Advantages of Nutrients and Biostimulants Derived from Sewage Sludge by Alkaline Thermal Hydrolysis Process for Agricultural Use: Quality Promotion of Soil and Crop
Source: Adv Sci (Weinh). 2024 Jan 19;11(13):2307793. doi: 10.1002/advs.202307793 (PMC10987130; doi:10.1002/advs.202307793)
Supplement: Supplementary file 1 — Supporting Information [file ADVS-11-2307793-s001.pdf]

ADVANCED SCIENCE
Open Access

Supporting Information

for *Adv. Sci.*, DOI 10.1002/advs.202307793

Double Advantages of Nutrients and Biostimulants Derived from Sewage Sludge by Alkaline Thermal Hydrolysis Process for Agricultural Use: Quality Promotion of Soil and Crop

Jiahou Hao, Bingbing Li, Jiayi Tan, Yue Zhang, Xuejia Gu, Shuo Wang, Yun Deng, Xiaokai Zhang and Ji Li**

Supporting Information

Double advantages of nutrients and biostimulants derived from sewage sludge by alkaline thermal hydrolysis process for agricultural use: quality promotion of soil and crop

Jiahou Hao[a], Bingbing Li[b], Jiayi Tan[a], Yue Zhang[c], Xuejia Gu[d], Shuo Wang[a,*], Yun Deng[a], Xiaokai Zhang[a], Ji Li[a,*]

[a] Jiangsu Key Laboratory of Anaerobic Biotechnology, School of Environment & Ecology, Jiangnan University, Wuxi 214122, China

[b] College of Life Sciences, Anhui Agricultural University, Hefei 230036, China

[c] China Civil Engineering Society Water Industry Association, Beijing 100082, China

[d] Heilongjiang Academy of Black Soil Conservation and Utilization, Harbin 150086, China

*Corresponding author at:

School of Environment & Ecology, Jiangnan University, Wuxi 214122, China

Email address: shuowang@jiangnan.edu.cn (S. Wang); liji@jiangnan.edu.cn (J. Li)

Text

Text S1. Detailed procedures for the determination of phytohormones and allelopathic substances.

A mixture of 500ul sample solution and 20 μL internal standard myristic-d27 acid (Sigma Aldrich, 366889) internal standard at a concentration of 0.3 mg•mL^{-1} was dissolved in 0.8 mL of aqueous methanol (v/v = 3:1). The mixed samples were vortexed for 5 min, sonicated at 60 Hz for 10 min, and stored at 4°C for 10 min, and centrifuged at 13500 g for 10 min at 4°C. 1000 μL of the supernatant was transferred to a glass injection vial and dried under vacuum at room temperature. Then 80 μL of methoxyamine hydrochloride (dissolved in pyridine at a concentration of 15 mg•mL^{-1}) was added, and the mixture was vortexed vigorously for 2 minutes and incubated at 37°C for 90 min. Next, 80 μL of N-Methyl-N-(trimethylsilyl) trifluoroacetamide (Aladdin, M122796) and 20 μL of n-hexane were added to the mixture, vortexed vigorously for 2 minutes, and then derivatized at 70°C for 60 minutes. Derivatized samples were analyzed by gas chromatography time of flight mass spectrometry (Pegasus BT, Leco, USA). The phytohormones and allelopathic concentrations of the selected spectra were then semi quantitatively calculated by direct comparison with the internal standard peak area.

Figure

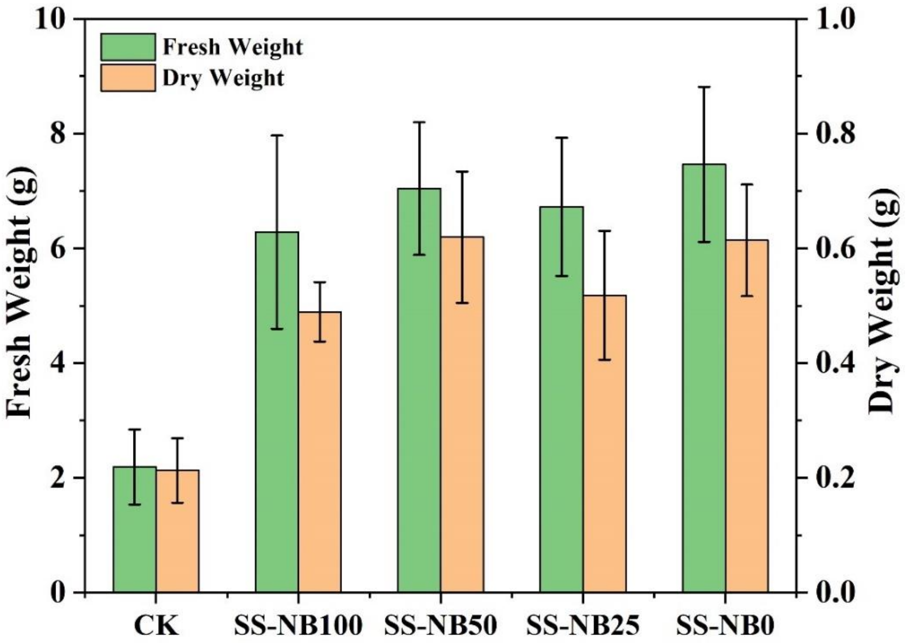

Figure S1. Crop yield (fresh weight and dry weight).

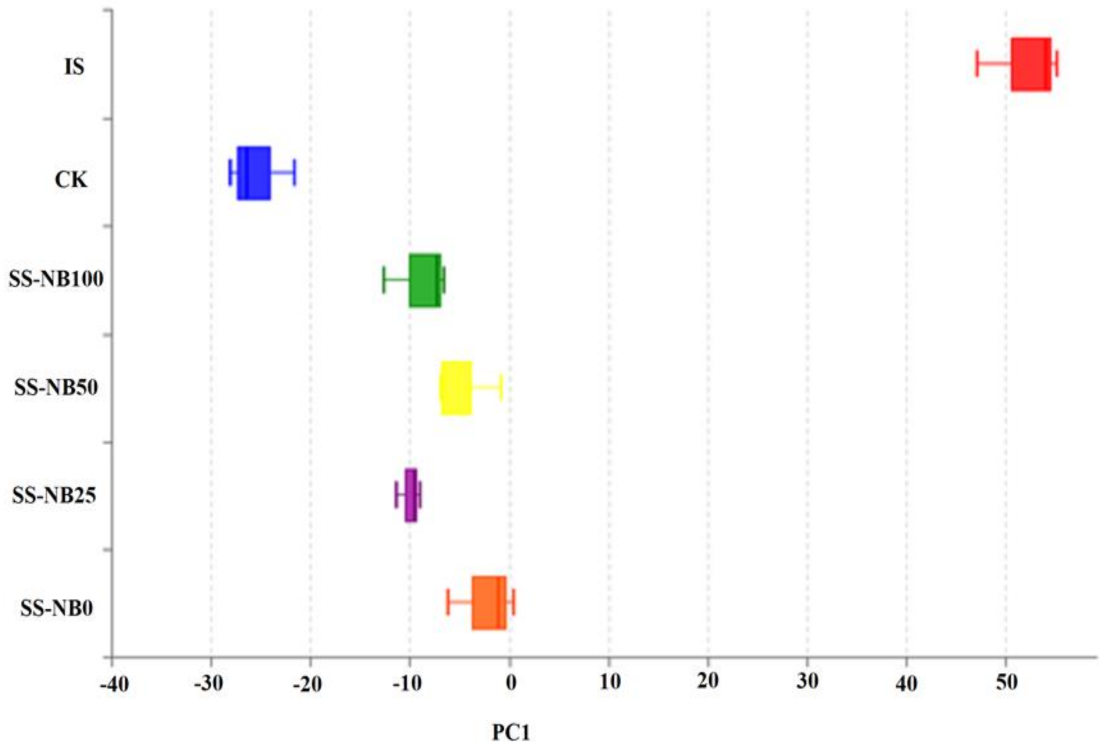

Figure S2. Dispersion of SS-NB100, SS-NB50, SS-NB25 and SS-NB0 on the PC1 axis.

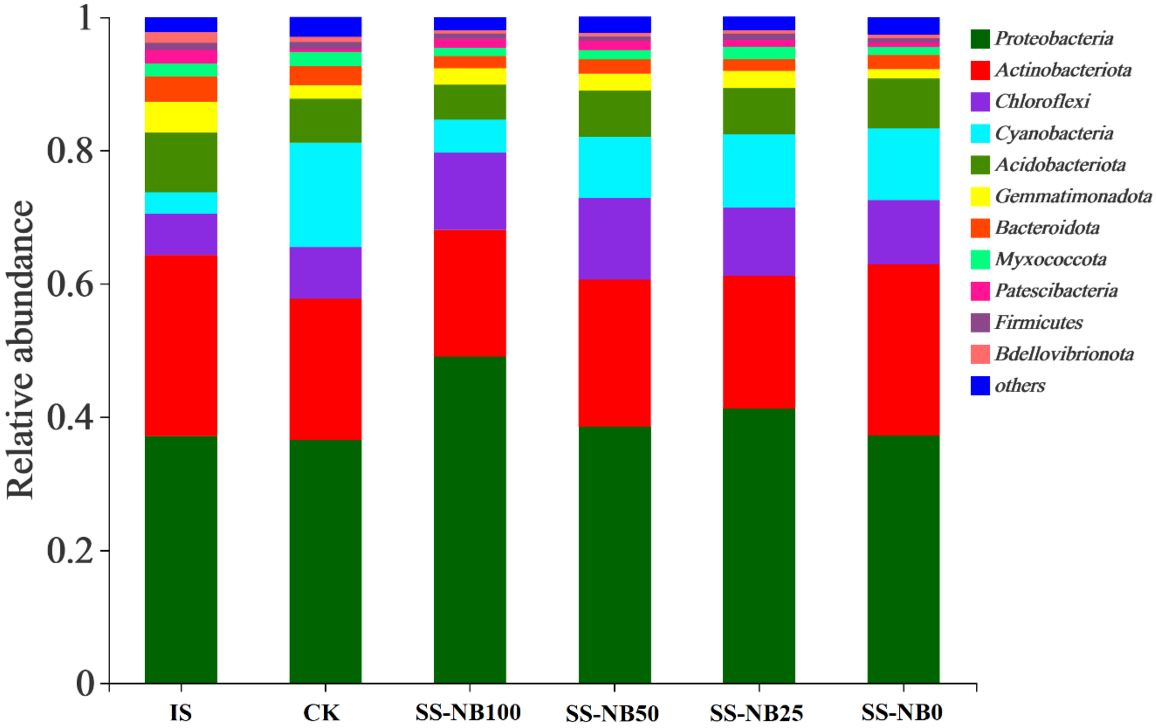

Figure S3. Bacterial community structures of Initial Soil (IS), CK, SS-NB100, SS-NB50, SS-NB25 and SS-NB0 at phylum level.

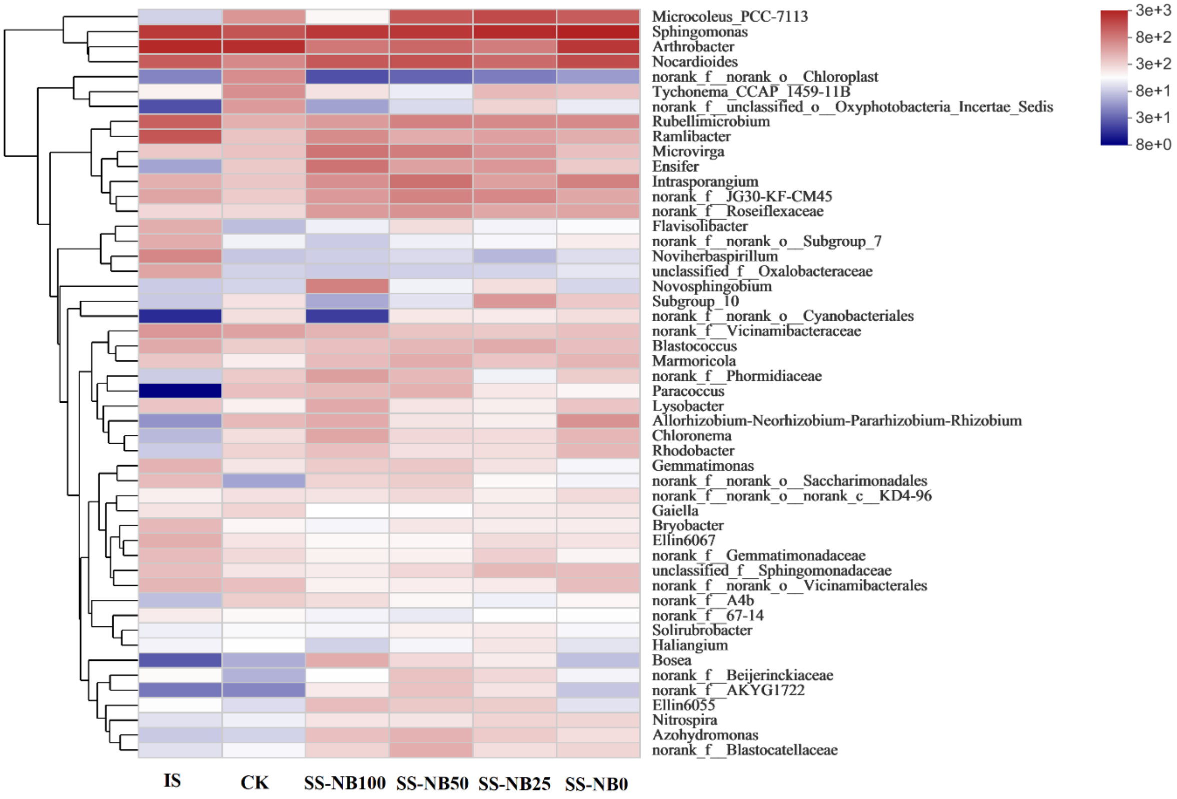

Figure S4. Microbial community heatmap analysis at genus level (top 50 for abundance).

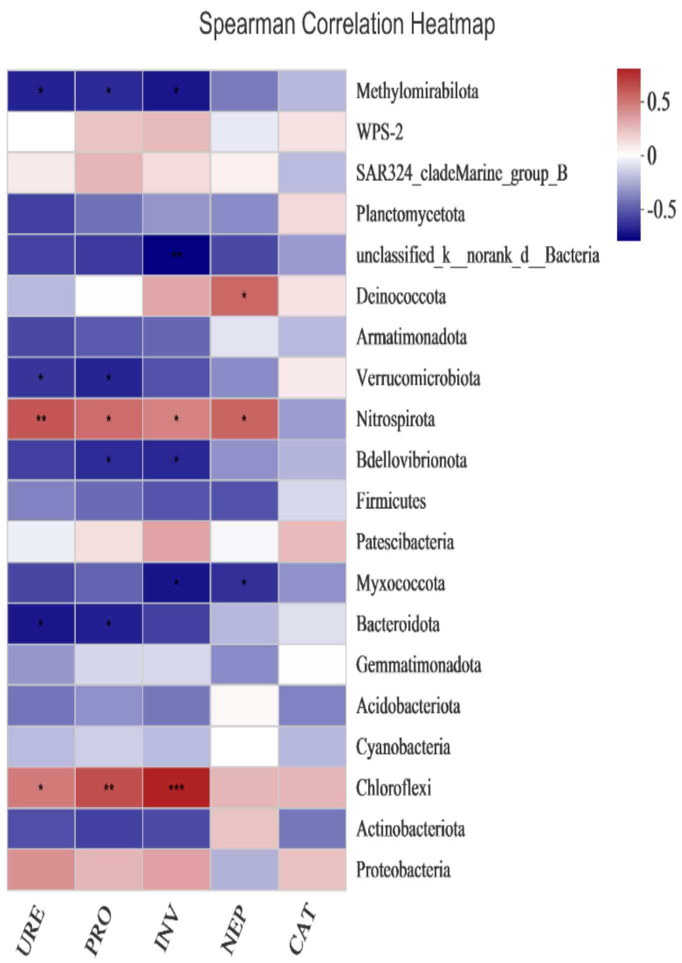

Figure S5. Correlation analysis between enzyme activity and microbial community at phylum level (top 20 for abundance).

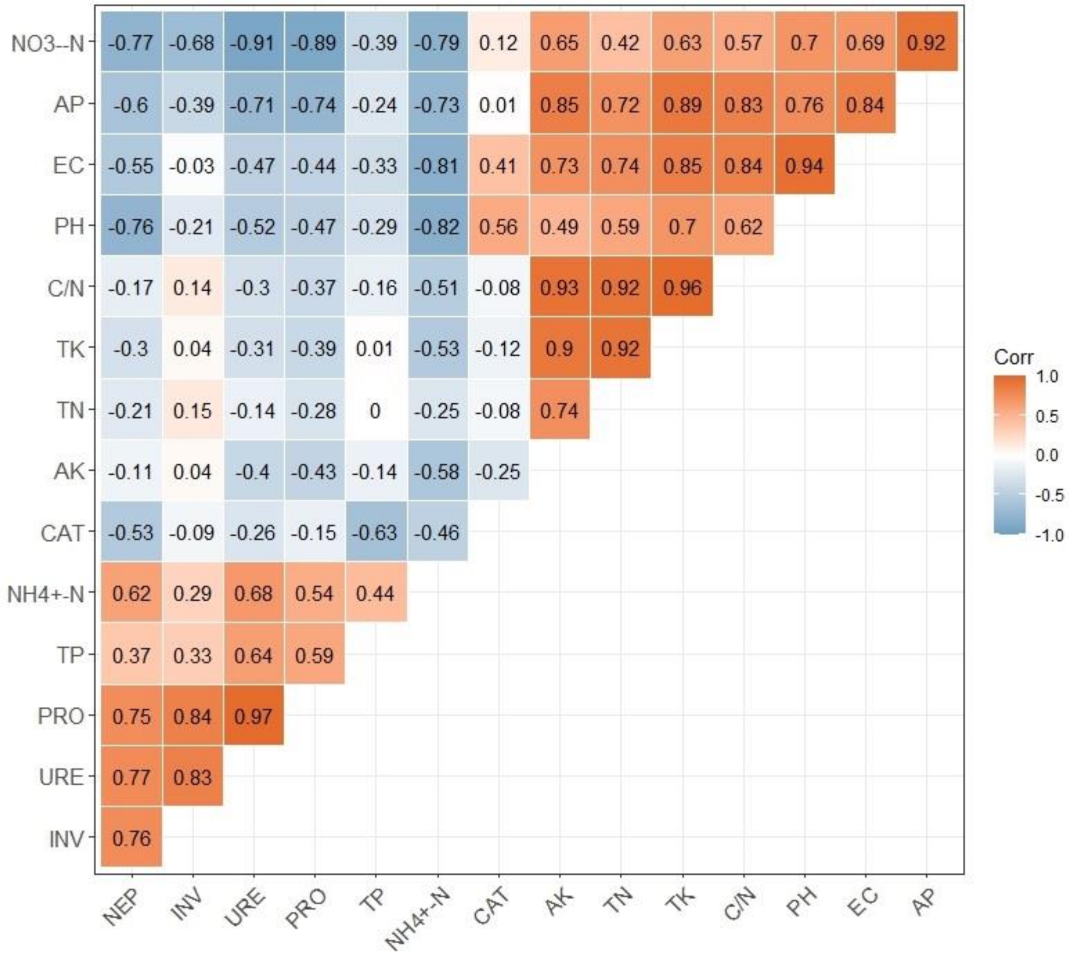

Figure S6. Heat map of correlation between soil physicochemical properties and enzyme activities.

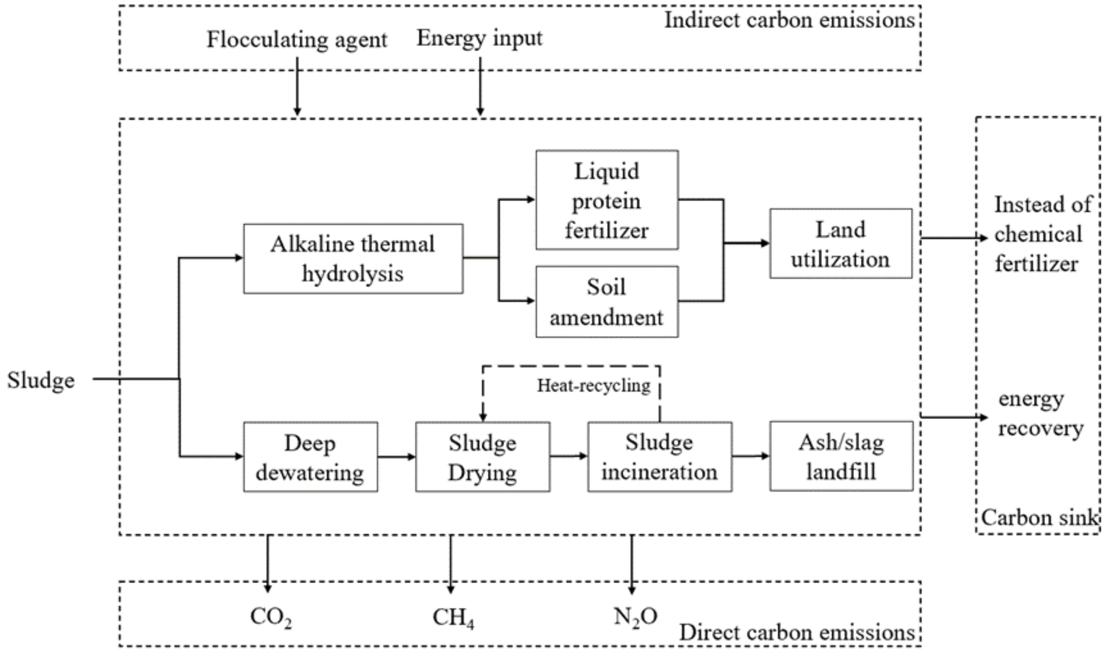

Figure S7. Total carbon emission accounting boundary.

Table

Table S1. Effects of SS-NB.

	pH	Electric conductivity ($ms \cdot m^{-1}$)	Soil C/N	Organic matter (g/kg)
IS	8.29±0.01	13.86±0.27	2.30±0.41	2.30±0.44
CK	8.11±0.11	11.64±0.18	1.04±0.26	1.55±0.09
SS-NB100	8.21±0.02	12.36±0.77	1.35±0.29	1.61±0.53
SS-NB50	7.99±0.02	11.69±0.22	1.19±0.31	1.37±0.53
SS-NB25	7.92±0.10	10.85±0.80	1.12±0.10	1.30±0.61
SS-NB0	7.72±0.14	10.16±0.24	1.19±0.15	1.18±0.61

Table S2. Richness and diversity of microbial community.

Treatment	Ace	Chao	Simpson	Shannon
CK	2706.98±80.11	2706.69±83.63	0.9886±0.0031	6.06±0.21
SS-NB100	2254.98±235.02	2278.06±176.53	0.9918±0.0010	5.76±0.21
SS-NB50	2346.58±18.31	2386.38±75.18	0.9872±0.0099	5.75±0.25
SS-NB25	2446.16±147.78	2428.95±86.19	0.9888±0.0048	5.84±0.27
SS-NB0	2324.57±46.71	2334.64±65.10	0.9842±0.0052	5.64±0.16

Table S3. Energy and material consumption parameters in sludge treatment and disposal process.

Items		Parameter
Deep dewatering	Power consumption of filtration	45 Kw·h/t H_2O
	Quick lime	300 kg/t DS
	Ferric chloride	70 kg/t DS
Sludge drying	Power consumption of drying	270 Kw·h/t H_2O
	Natural gas consumption	3.024 GJ/t H_2O
Sludge incineration	Power consumption of incineration	400 Kw·h/t H_2O
	Natural gas consumption	0.436 GJ/t H_2O
	Calorific value of raw sludge	12.55 KJ/Kg DS
	Calorific value after conditioning with CaO	7.48 KJ/Kg DS
	Heat loss rate	30%
Ash/slag landfill	Power consumption	12.5 Kw·h/t DS
	Diesel consumption	1.7 kg /t DS
Alkaline thermal hydrolysis	Power consumption	406 kW·h/t DS
	Steam consumption	1.37 t/t DS
	Quick lime	200 kg/t DS
	Sulfuric acid	20 kg/t DS
	Yield of liquid protein fertilizer	250 kg/t DS
	Crude protein content of liquid protein fertilizer	30%
	Yield of soil amendment	1.55 t/ t DS (40% moisture content)
	Crude protein content of soil amendment	8%
Sludge transportation	Transport distance	10 km
	Vehicle load	10 t
	Diesel consumption (Full-load)	0.255 kg/km
	Diesel consumption (no-load)	0.153 kg/km

Table S4. Emission Factors of CO_2.

Items	Carbon dioxide emission factor
Quick lime	8.3 kgCO_2/kg
Ferric chloride	1.74 kgCO_2/kg
Sulfuric acid	2.5 kgCO_2/kg
Nutrient solution	2.5 kgCO_2/kg
Electricity	0.7478 kgCO_2/kWh
Natural gas	56.1 kgCO_2/GJ
Steam	0.0861 kgCO_2/kg
Diesel	3.186 kgCO_2/L
Fertilizer	0.71 kgCO_2/kg
